# Saliva as a Future Field in Psoriasis Research

**DOI:** 10.1155/2018/7290913

**Published:** 2018-05-20

**Authors:** Farah Asa'ad, Marco Fiore, Aniello Alfieri, Paolo Daniele Maria Pigatto, Chiara Franchi, Emilio Berti, Carlo Maiorana, Giovanni Damiani

**Affiliations:** ^1^Department of Biomedical, Surgical & Dental Sciences, University of Milan, 20122 Milan, Italy; ^2^Department of Women, Child and General and Specialized Surgery, University of Campania “Luigi Vanvitelli”, 80138 Naples, Italy; ^3^Clinical Dermatology, Department of Biomedical, Surgical and Dental Sciences, IRCCS Galeazzi Orthopaedic Institute, University of Milan, 20126 Milan, Italy; ^4^Dipartimento di Fisiopatologia Medico-Chirurgica e dei Trapianti, Universita' degli Studi di Milano, Unita' Operativa di Dermatologia, IRCCS Fondazione Ca' Granda, Ospedale Maggiore Policlinico, 20122 Milano, Italy; ^5^Center for Jawbone Atrophies Policlinico Hospital, University of Milan School of Dentistry, 20122 Milan, Italy; ^6^Young Dermatologists Italian Network (YDIN), GISED, 24122 Bergamo, Italy

## Abstract

Psoriasis is a skin inflammatory disease characterized by an increased body of comorbidities, including parodontopathy. Despite the visibility of skin lesions, prognostic biomarkers, related to disease monitoring and therapeutic effectiveness, are still missing. Although several markers have been studied, none of them has been identified as an independent prognostic factor. This concise review aims to summarize the current knowledge and results in saliva research applied to psoriasis. Combination of different markers could improve the prognostic prediction in patients with psoriasis. Future studies are needed to implement research on salivary biomarkers and their prognostic/therapeutic effects in the management of patients with psoriasis.

## 1. Psoriasis and the Need of New Biomarkers

**Figure 1:**
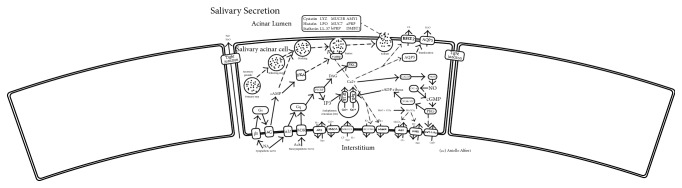
The two salivary secretory pathways: protein exocytosis and fluid secretion. Salivary secretion is modulated by the autonomous system: the sympathetic system activates the adenylate cyclase increasing the level of cAMP leading to the secretion of proteins from the secretory granules. The parasympathetic, instead, by the upmodulation of phospholipase C causes the raising of intracellular Ca2+ and fluid secretion. BEST2: bestrophin 2. AQP5: aquaporin 5. VAMP: vesicle associated membrane protein. CALM: calmodulin like 6. CD38/157: bone marrow stromal cell antigen 1. PKG: protein kinase, cGMP-dependent, type I. GC-s: guanylate cyclase 1 soluble subunit alpha 2. NOS: nitric oxide synthase 1. RYR: ryanodine receptor 3. IP3r: inositol 1,4,5-trisphosphate receptor type 1. Cat-like: transient receptor potential cation channel subfamily V member 6. NHE1: solute carrier family 9 member A1. MaxiK: potassium large conductance calcium-activated channel subfamily M alpha member 1. KCNN4: potassium intermediate/small conductance calcium-activated channel subfamily N member 4. NKCC1: solute carrier family 12 member 2. PMCA: ATPase plasma membrane Ca2+ transporting 1. ATP: ATPase Na+/K+ transporting family member beta 4. M3R: cholinergic receptor muscarinic 3. *α*1R: adrenoceptor *α* 1D. AC: adenylate cyclase 1. *β*R: adrenoceptor beta 1. Gs: guanine nucleotide-binding protein G(s) subunit *α*. Gq: guanine nucleotide-binding protein G(q) subunit *α*. PLC*β*: phospholipase C *β* 1. MUC5B: mucin 5B, oligomeric mucus/gel-forming. MUC7: mucin 7, secreted. AMY1: amylase, alpha 1A. aPRP: proline rich protein HaeIII subfamily 1. bPRP: proline rich protein BstNI subfamily 1. DMBT1: deleted in malignant brain tumors 1 protein. Cystatin: cystatin-SN. Histatin: histatin 1. Statherin: statherin. LYZ: lysozyme C. LPO: lactoperoxidase. LL-37: cathelicidin antimicrobial peptide. See [12–14].

Psoriasis is an immune-mediated systemic inflammatory skin disease, interpreted as complex, because of the genetic, immunological, and environmental aspects [1–5]. The clinically visible manifestations are objectivized on the skin where well demarcated, infiltrated, and peripherally erythematous skin plaques are present, due to altered differentiation and hyperproliferation to the keratinocytes together with multifarious inflammatory cells and neoangiogenesis [6, 7]. An increased body of evidence has linked psoriasis to several comorbidities such as cardiovascular and periodontal ones and suggested the need for an affordable set of biomarkers [8, 9]. Currently, many biomarkers were proposed for psoriasis; however, none of them was considered as a valid and accepted disease marker. The ideal biomarker is a biological hallmark that is sensitive, specific, reproducible, and capable of identifying a physiological or pathological status and/or a therapeutic response [10]. Furthermore, the biomarker assay should be validated, standardized, and easy to perform [10]. In the past years, the biomarkers research in psoriasis focused on assessing blood and skin samples, genetics, and transcriptomics with contrasting results [11]. Therefore, saliva with its two secretory pathways has gained growing interest as an alternative and available biological sample to analyze, looking for biomarkers (Figure 1) [12–14]. Available evidence, first maturated in the field of rheumatic diseases, suggests the relation between saliva, oral inflammation, and systemic health. In addition, oral microbiome is also related to both skin and gastrointestinal system, consequently modulating the systemic inflammation [15]. This concise review summarizes the current evidence regarding the applications of saliva in psoriasis and describes future approaches in the field.

## 2. Salivary Changes in Rheumatic Diseases

**Figure 2:**
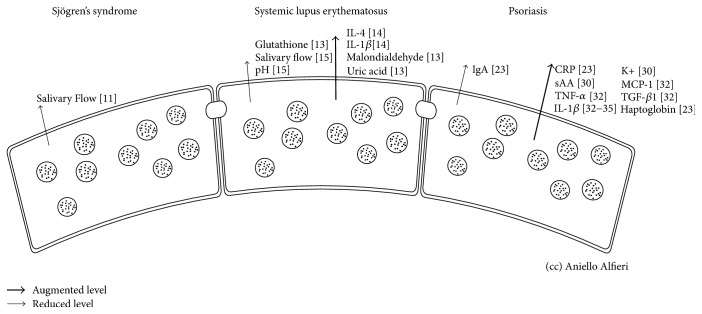
Component changes of salivary secretion in rheumatic diseases and psoriasis. sAA: alpha amylase; K+: potassium; CRP: C-reactive protein; TNF: tumor necrosis factor; TGF: transforming growth factor; MCP: monocyte chemoattractant protein; IL: interleukin.

Several rheumatic diseases may compromise the physiological salivary function (Figure 2), causing acute and chronic disorders. They are analyzed in detail in Table 1. The most common oral changes in rheumatic diseases are hyposalivation (low salivary flow) and xerostomia. In Sjögren's syndrome, the lymphocytic infiltration of salivary glands can lead to secretory hypofunction leading to xerostomia [16]. The progression of lymphocytic infiltration gives rise to focal sialadenitis, salivary gland dysfunction, and, in about quarter of patients, an expansion of the parotid and submandibular glands, which are often associated with a reduced salivary flow [16]. Furthermore, the reduced antimicrobial effect of the salivary secretion and the poor lubrication of the mouth facilitate the onset of oral infections, mucosal fragility, and burning mouth syndrome (BMS). Other complications include oral candidiasis, dental caries, dysphagia, and dyspepsia [17]. In systemic lupus erythematosus (SLE), saliva was demonstrated to be an incredible source of biomarkers (Table 1), revealing an impaired antioxidant power with reduced levels of glutathione and conversely high levels of malondialdehyde and uric acid [18]. This data was also confirmed by the high levels of proinflammatory salivary cytokines IL-1*β* and IL-4, which are also positive markers to periodontal disease [19]. Thus, the high levels of caries found in SLE patients might be justified by the inflammatory microenvironment of the oral cavity and by the decrease in salivary flow, pH, and buffer capacity [20]. The Behçet disease, instead, is correlated with dysbiosis of the salivary microbiota, probably implicated in recurrent aphthous stomatitis [21]. Xerostomia is also common in bullous pemphigoid and in mucous membrane pemphigoid. Despite a correlation between BP-180 salivary levels being discouraged in bullous pemphigoid [22], the correlation between skin disease severity, the Autoimmune Bullous Skin Disorder Intensity Score (ABSIS), and salivary levels of both desmogleins 1 and 3 remains a matter of debate in pemphigus vulgaris [22, 23]. Furthermore, saliva suggested a possible connection between herpes simplex and pemphigus vulgaris; Ruocco et al. detected herpes simplex DNA in very early stages of pemphigus vulgaris, leading to the hypothesis of a potential viral role in triggering the autoimmune response [24].

## 3. Saliva and Biochemical Characteristics in Psoriasis

In many studies, different salivary components have been evaluated in psoriasis patients (Table 2), such as salivary total protein, salivary immunoglobulin A (IgA), IgG, lysozyme, C-reactive protein (CRP), and Haptoglobin. However, findings have been inconclusive for some markers due to contradictory results between studies, which might be attributed to the difference in the type of investigated psoriasis [29–31]. Although Krasteva and colleagues reported no statistically significant difference in the salivary level of IgA, assessed by radial immunodiffusion, between psoriasis patients and healthy controls, they observed that patients with a PASI > 10 had a tendency to show lower levels of IgA, compared to patients with a PASI < 10, suggesting that patients with a PASI > 10 might be at high risk of developing microbial infections that could trigger psoriasis [25]. In the same study, the authors reported a statistically significant increase of salivary C-reactive protein (CRP), associated with the inflammatory nature of the psoriasis, and, as known, CRP has a prognostic significance for the worsening of psoriasis [32]. Similarly, increased salivary levels of Haptoglobin were reported, indicating a local defense mechanism against psoriasis [25]. The levels of salivary CRP and Haptoglobin were determined by an immunoturbidimetric method.

In another study, Soudan and coworkers assessed the alterations of other salivary components and their correlation with the severity of psoriasis [33]. Sodium (Na+), potassium (K+), chloride (Cl−), and alpha amylase (sAA) were analyzed using ISE (Ion Selective Electrode) technology for electrolyte measurements and LISA 500 plus systems for sAA. Findings showed a significantly higher K+ and sAA concentrations in psoriasis patients, with respect to controls, while there was no significant rise in the other investigated salivary ions. Nonetheless, these changes were not related to either the severity or the duration of psoriasis. Recently, Bottoni et al. analyzed the saliva proteomic components in psoriatic patients against diabetic patients and healthy controls, by attenuated total reflection (ATR) in conjunction with infrared spectroscopy. There were differences in the secondary structure composition of proteins between psoriatic and diabetic patients as compared to the control group [26]. Moreover, the authors concluded that the saliva spectra of the control group and that of the palmoplantar psoriatic patients differ from plaque psoriasis and diabetic patient spectra because of the absence of the amide II band and the presence of different secondary protein-structure conformations. Although all the previous findings suggest the difference in biochemical components between psoriasis patients and healthy individuals, and also between different psoriatic disease entities, more studies are required to support the preliminary results in literature.

## 4. Saliva and Correlated Biomarkers in Psoriasis

Although salivary biomarkers have been identified in various systemic diseases, evidence is still scarce on the biomarkers that appear in the saliva and are correlated to psoriasis [34, 35]. In this context, Ganzetti et al. evaluated the expression levels of interleukin- (IL-) 1*β*, IL-6, transforming growth factor- (TGF-) *β*1, IL-8, tumor necrosis factor- (TNF-) *α*, interferon- (IFN-) c, IL-17A, IL-4, IL-10, monocyte chemoattractant protein- (MCP-) 1, macrophage inflammatory protein- (MIP-) 1*α*, and MIP-1*β* in salivary secretions from patients with psoriasis, using multianalyte ELISA Arrays [27]. The authors reported a statistically significant greater expression of TNF-*α*, TGF-*β*1, MCP-1, and IL-1*β* in saliva of patients with psoriasis than in healthy subjects, with a positive correlation between IL-1*β*, TGF-*β*1, and MCP-1 expression and oral disease severity [27]. In fact, it was suggested that the increased expression of IL-1*β* in patients with psoriasis might explain why such individuals show more missing teeth and more alveolar bone resorption and periodontitis than healthy individuals, as previously reported [36]. Periodontitis is a destructive disease of the tooth-supporting tissues induced by bacterial biofilm [37] and, in highlight of the reported findings, it seems that psoriasis and periodontal disease share the underlying inflammatory process, especially that IL-1*β* could be responsible for tissue destruction in periodontal disease by increasing the levels of matrix metalloproteinases [38]. Higher IL-*β* salivary levels in psoriasis with respect to healthy individuals were supported in another study; Mastrolonardo et al. observed higher basal IL-1*β* levels among psoriatic patients suggesting an increase in its production [28]. Such changes in cytokine activity may play an important role in propagating inflammation in psoriatic skin. Furthermore, the association between psoriasis and oral mucosa could be indicated by the increased salivary [27] and serum levels of TGF-*β*1 and MCP-1 in psoriasis patients [39, 40]. In a different investigation, Ganzetti and coworkers confirmed the validity of saliva as a noninvasive tool to monitor inflammation in psoriasis [41]. At baseline, psoriasis patients had higher salivary levels of IL-1*β*, evaluated via an enzyme-linked immunosorbent assay (ELISA), in comparison to healthy individuals. After 12 weeks of treatment with TNF-*α* inhibitors, IL-1*β* levels significantly reduced in comparison to that of baseline, but remained significantly higher than in healthy controls even after treatment. Nonetheless, larger cohort studies are still needed to confirm these findings.

## 5. Saliva as a Monitor for Antipsoriatic Drugs

For more than four decades, it was possible to predict plasma concentrations of drugs by measuring the salivary drug concentration, avoiding the need of venipuncture [42]. This is especially desirable in patients within the pediatric and geriatric population, in which venipuncture could be difficult to perform. The prediction of plasma concentration is mostly reliable if there is a documented correlation between plasma drug levels and clinical outcomes. In fact, this concentration is mostly reliable for nonionized drugs at normal plasma pH (phenytoin, phenobarbital, and antipyrine) but is unreliable for ionized drugs (chlorpropramide, tolbutamide, propranolol, and meperidine). In clinical practice, there is scarce evidence indicating whether this strategy is useful for antipsoriatic drugs. Pediatric patients with rheumatological diseases receiving methotrexate chronically have shown poor correlation between serum methotrexate concentrations and salivary levels [43]. Also, in a study that enrolled adults with various malignant diseases, the measurements of methotrexate salivary concentrations have shown no correlation with serum methotrexate levels, in monitoring patients after 24 hours of methotrexate infusions [44]. More recently, a sensitive and a simple quantifying method by liquid chromatography-tandem mass spectrometry (LC-MS/MS) was shown to be a valid method for determination of saliva excretion on samples obtained after an intravenous administration of 1 mg/kg/dose of methotrexate to six patients with acute lymphoblastic leukemia [45]. This method could be a promising tool for the quantification of methotrexate in the saliva of patients with psoriatic disease as well. The LC-MS/MS method has been also investigated for the measurement of cyclosporine in saliva and a correlation between the cyclosporine concentrations in fifteen paired blood-saliva samples from kidney transplant recipients was shown to be significant [46]. The method of saliva collection coupled with the LC-MS/MS quantification technique for cyclosporine analysis could be also beneficial if applied to psoriatic patients undergoing cyclosporine therapy. Less encouraging results were reported with the monoclonal fluorescent polarization immunoassay (FPIA) kit, adapted to salivary testing by using a novel extraction method developed and patented under the name of Middle East Research Institute (MERI) [47]. This method showed no significant correlation between blood and salivary cyclosporine levels [47]. Psoraderm 5, Meladinine, and Oxsoralen, three psoralens utilized during PUVA therapy, were well monitored with salivary sampling. This method is a noninvasive alternative to serum levels measurements and is suitable for routine applications; five or six salivary samples are sufficient to determine tmax in a patient starting photochemotherapy, while three samples are mandatory for Cmax [48]. Steroids, often prescribed for psoriasis treatment, may cause adrenocortical suppression; as a screening technique for iatrogenic adrenal suppression, morning salivary cortisol (MSC) has 100% sensitivity and 97% specificity [49]. MSC assay is an accurate tool for monitoring adrenal function and should be recommended for psoriatic patients who are on steroid therapy.

The techniques reviewed are well established methodologies for therapeutic drug monitoring (TDM). High-performance liquid chromatography (HPLC), capillary electrophoresis (EC), liquid chromatography (LC), gas chromatography (LC or GC) coupled with UV spectroscopy, or mass spectrometry (MS) has been extensively applied to TDM, optimizing protocols for the analysis of drugs administered and the analysis of drug metabolites if necessary. Compared to sophisticated and large-scale techniques, like high-performance liquid chromatography-mass spectrometry (HPLC-MS), immunoassays are easily and readily miniaturized by employing nanostructured surface sensors or micro- and nanoparticles (NP), and microfluidic versions of these analytical devices are already available for research purposes. Recent advancements in point-of-care (PoC) technologies show great transformative promises for personalized preventive and predictive medicine. However, fields like TDM, which first allowed for personalized management of patients' disease, are still lacking in the widespread application of PoC devices [50].

Future studies may be warranted to design PoC technologies, more reliable and less invasive procedures, for therapeutic antipsoriatic drug monitoring.

## 6. Future Perspectives

Salivary biomarkers levels tend to change in psoriasis (Figure 2), which reflect alterations in the their production/expression. Although salivary biomarkers in psoriasis patients have been evaluated in different studies, these investigations were only of a small sample size and focused only on specific biomarkers. Therefore, studies with wider salivary profiling and larger sample size are needed to confirm the preliminary findings in literature. Moreover, studies monitoring the salivary level changes of these biomarkers after psoriasis treatment are recommended, as more research on potential salivary prognostic biomarkers in psoriasis patients is still needed. In fact, salivary biomarkers might be helpful detectors of psoriasis severity and disease progression and could serve as valuable diagnostics in the future.

## Figures and Tables

**Table 1:** Changes of salivary function and components in patients with rheumatic diseases. IL: interleukin.

Disease	Salivary change	Effect
Sjögren's syndrome	Salivary flow	↓[16]

Systemic lupus erythematosus	Glutathione	↓[18]
	Malondialdehyde	↑[18]
	Uric acid	↑[18]
	IL-1*β*	↑[19]
	IL-4	↑[19]
	Salivary flow	↓[20]
	pH	↓[20]

**Table 2:** Changes of salivary function and components in patients with psoriasis.

Disease	Salivary change	Effect
Psoriasis	IgA^*∗*^	↓[25]
	CRP	↑[25]
	Haptoglobin	↑[25]
	K+	↑[26]
	sAA	↑[26]
	TNF-*α*	↑[27]
	TGF-*β*1	↑[27]
	MCP-1	↑[27]
	IL-1*β*	↑[27, 28]

^*∗*^PASI > 10; sAA: alpha amylase; K+: potassium; CRP: C-reactive protein; TNF: tumor necrosis factor; TGF: transforming growth factor; MCP: monocyte chemoattractant protein; IL: interleukin.
